# The Propensity for Inducing Atrial Fibrillation: A Comparative Study on Old versus Young Rabbits

**DOI:** 10.1155/2014/684918

**Published:** 2014-03-02

**Authors:** Hongliang Li, Benjamin J. Scherlag, David C. Kem, Caitlin Zillner, Shailesh Male, Sorkko Thirunavukkarasu, Xiaohua Shen, Alexandria Benbrook, Jan V. Pitha, Ralph Lazzara, Xichun Yu

**Affiliations:** ^1^Heart Rhythm Institute, Department of Medicine, University of Oklahoma Health Sciences Center and Veterans Affairs Medical Center, Oklahoma City, OK 73104, USA; ^2^Department of Pathology, University of Oklahoma Health Sciences Center and Veterans Affairs Medical Center, Oklahoma City, OK 73104, USA

## Abstract

It is well established that atrial fibrillation (AF) is far more common in elderly humans. Autonomic activation is thought to be an operative mechanism for AF propensity. The aim of the study was to investigate the impact of age on atrial tachyarrhythmia induction in a rabbit model. Six old (aged 4–6 years) and 9 young (aged 3-4 months) New Zealand white rabbits were subjected to a catheter-based electrophysiological study. Atrial tachyarrhythmia susceptibility was tested by burst pacing before and after infusion of increasing concentrations of acetylcholine. Both young and old rabbits were in normal sinus rhythm at the beginning of the infusion/burst pacing protocol. The old rabbits had faster heart rates and a marked increase in atrial tachyarrhythmias compared to the young rabbits. Nonsustained and sustained AF events were more frequent in the old rabbits. No significant fibrosis was observed in the atria of either young or old rabbits. In conclusion, the old rabbits have a greater propensity for induction of AF. The significantly faster heart rates in the old rabbits suggest that dominant sympathetic activity may play an important role in the propensity for AF in this group.

## 1. Introduction

The incidence of atrial fibrillation (AF) in the human population increases with age, particularly in those over the age of 60 [1]. The mechanisms proposed for contributing to this association have ranged from structural [2, 3] and electrophysiological [4, 5] to metabolic issues [6–8]. The well-known association of AF with thyrotoxicosis has long been associated with older age [6–8]. We have previously reported a group of patients with thyrotoxicosis and concurrent AF and confirmed the interaction of increasing age as well as the significant association and effect of autoantibodies that activate the *β*-adrenergic and M2 muscarinic receptors [9]. In a previous study, we infused the autonomic neurotransmitter acetylcholine (ACh) into rabbits that had been immunized to produce activating autoantibodies to the *β*2-adrenergic receptor [10]. The results indicated a significantly greater incidence of sustained atrial tachycardia than in the same rabbits prior to immunization. In preliminary studies in selected animals that were older than 4 years of age, we observed a much lower threshold for induction of atrial tachyarrhythmias (unpublished observations). For this reason, we have investigated whether an autonomic mechanism may be the basis for a greater AF propensity in an old (4–6 years) group of rabbits compared to young (3-4 months) rabbits in whom different concentrations of ACh were infused intravenously to test the inducibility of AF with concomitant burst atrial pacing.

## 2. Materials and Methods

### 2.1. In Vivo Electrophysiological Assessment of Cardiac Tachyarrhythmia Propensity

**Figure 1:**
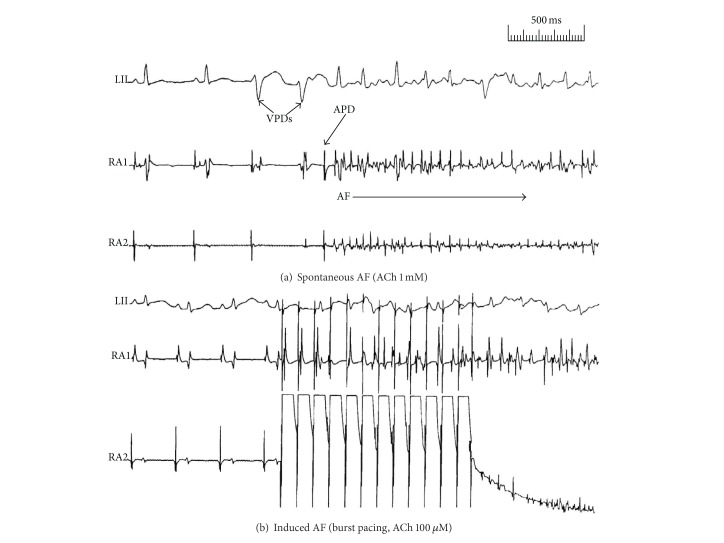
Examples of sustained atrial fibrillation (AF) in the old rabbits. (a) Spontaneously occurring sustained AF initiated by an atrial premature depolarization (APD) following two ventricular premature depolarizations (VPDs). (b) Burst pacing-induced sustained AF.

This study protocol was approved by the Institutional Animal Care and Use Committee of the Oklahoma City Veterans Affairs Medical Center and University of Oklahoma Health Sciences Center. Six old (aged 4–6 years) and 9 young (aged 3-4 months) New Zealand white rabbits of either sex fed standard rabbit chow were studied. Each animal was anesthetized with ketamine/xylazine (35 mg/5 mg/kg) and subjected to a catheter-based electrophysiologicalstudy [10]. Standard electrocardiograms (Leads 1-aVF) were continuously monitored. After shaving the neck area, the right jugular vein was dissected and cannulated with a 4-French multielectrode catheter. Under electrographic control, the catheter was passed into the right atrium to record atrial potentials in conjunction with the ECG leads. After a 5-minute stabilization and determination of the mean sinus rate, atrial tachyarrhythmia susceptibility was tested by delivering bursts of stimuli (3–5 sec duration) at a high frequency (20 Hz) and voltage that was at least twice the diastolic pacing threshold. Burst pacing was delivered 3–10 times before and after the infusion of ACh in 3 incremental concentrations (10 *μ*M, 100 *μ*M, and 1 mM) at a rate of 1 mL/min. Nonsustained (<10 sec) and sustained (≥10 sec) arrhythmia occurrence was determined in response to burst pacing at baseline and with each of the 3 concentrations of ACh infusions for 2 minutes before initiating burst pacing. Rhythm disturbances were characterized from the ECG by their rate and duration and from the morphology of the recorded atrial electrograms (Figure 1).

### 2.2. Histological Studies

Portions of the atria and ventricles from the hearts fixed in formalin were prepared for histological study. Specimens were processed in paraffin-embedded histological sections cut along the long axis of the atria and ventricles. Sections were stained with hematoxylin-eosin and Masson's trichrome for detection of fibrosis. The degree of fibrosis was quantified on the basis of the following scale: 0, no fibrosis; 1+, stainable collagen adjacent to capillaries and/or between myocytes (excluding large vessels) less than half of myocyte diameter; 2+, greater than half to equal to myocyte diameter; 3+, greater than myocyte diameter or fibrosis with features of replacement.

### 2.3. Statistical Analysis

Data are expressed as mean ± standard deviation (SD). A standard *t*-test was used to compare heart rates between young and old rabbits. Chi-square analysis (2 × 2 contingency table) followed by a two-tailed Fisher's exact was used to determine the difference in occurrence of a sustained arrhythmia (≥10 sec) for each rabbit at all concentrations of ACh infusion. A *P* value of <0.05 was considered statistically significant.

## 3. Results

In the baseline state, the mean heart rate for the old rabbits (*n* = 6) was 200 ± 23/min, compared to the young rabbits (*n* = 9) whose heart rate averaged 160 ± 13/min (*P* = 0.003). In addition, there were a significantly greater number of AF events in the old versus young rabbits (Table 1). There were more nonsustained (<10 sec) and sustained (≥10 sec) AF events in the old rabbits than the young rabbits. Specifically, in the old rabbits there were 13 nonsustained or sustained atrial tachyarrhythmias, either atrial tachycardia or AF induced in 24 induction attempts. On the other hand, the young rabbit group had only 4 such events in 36 induction attempts (*P* = 0.0004). As for sustained AF, there were 7 of 24 inductions in the old rabbits and 0 of 36 in the young rabbits (*P* = 0.0009). The average duration of AF in the old rabbits was 258 seconds. Figure 1 shows examples of the occurrence of spontaneous and pacing-induced AF in the old rabbits.

**Figure 2:**
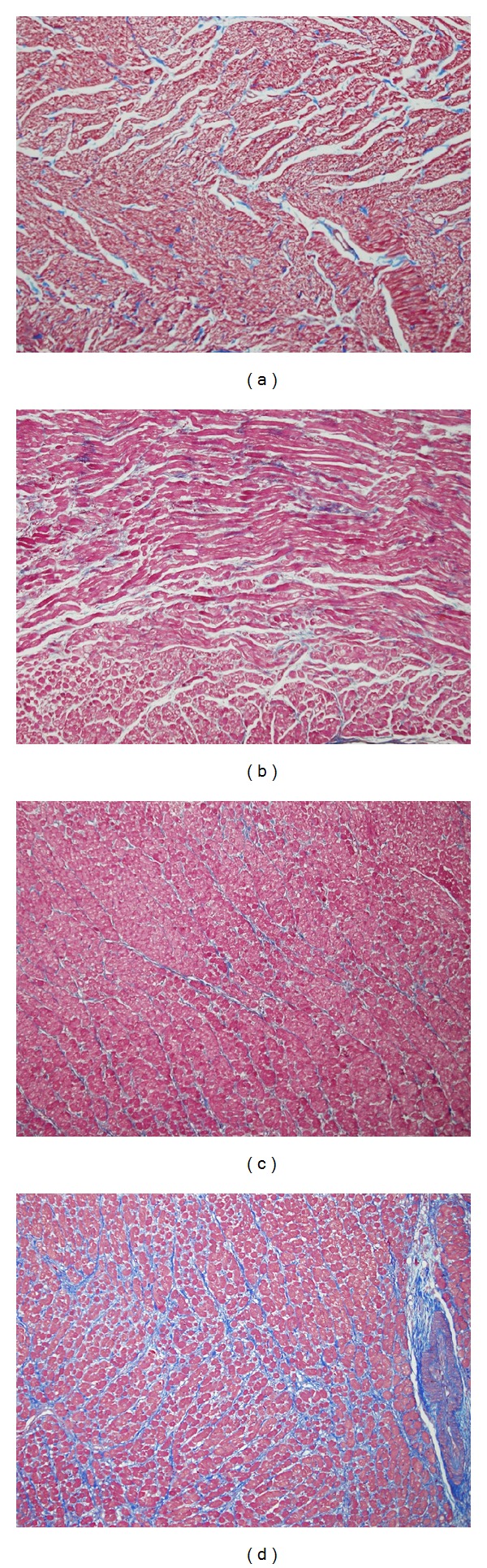
Representative histological sections of atria and ventricles from old and young rabbits. (a) Atrial cross section from a young rabbit (3-4 months). (b) Atrial cross section from an old rabbit (4–6 years). Neither atrial section showed appreciable interstitial fibrosis. (c) Ventricular cross section from a young rabbit. (d) Ventricular cross section from an old rabbit. Note the marked interstitial fibrosis and scar compared to (c).

To determine whether atrial fibrosis was a factor in the differences in AF susceptibility between old and young rabbits, we examined histological sections of the atria in both groups. In order to quantitate the degree of fibrosis, a grading system was developed. In the sections evaluated in the young rabbits, fibrosis was graded as 0 in four sections of atria and ventricular tissues, whereas in the old rabbits the grades for the atria ranged from 0 in 4 sections to 1+ in 3 sections. In the ventricles fibrosis was graded as 1+ in one section, 2+ in two sections, and 3+ in 3 sections. Figures 2(a) and 2(b) show representative atrial sections from the young versus old rabbits, respectively. There was no significant fibrosis observed in the atria of either group. However, the old rabbits did demonstrate moderate to severe interstitial fibrosis as well as scar formation in the ventricles (Figure 2(d)). There was no significant fibrosis seen in the ventricular sections made from the young rabbits (Figure 2(c)).

## 4. Discussion

In the present study, two extraneous factors (ACh infusion and burst pacing) were used to initiate arrhythmias. Previous studies in ambulatory dogs with a propensity for inducing atrial tachyarrhythmias were based on the simultaneous interaction of the neural activity of both arms of the autonomic nervous system [11]. In this rabbit model, there was a significantly greater incidence of AF, particularly of the sustained (≥10 sec) form, in the old rabbits than in the young group. The young rabbits showed no evidence of sustained AF using the same protocol of ACh infusions that were effective in inducing AF in the old rabbits. Lee et al. [12] previously reported that 3-year-old rabbits, fed a standard chow, and a second comparably aged group fed a high cholesterol chow demonstrated a greater atrial tachyarrhythmia response to programmed stimulation and/or rapid atrial pacing than the 3-month-old rabbits fed a standard chow. The maximal duration of the atrial arrhythmias in this study was 30 seconds and consisted predominantly of atrial tachycardias. In the present study, infusion of incremental concentrations of ACh and burst pacing consistently induced sustained AF with a mean uninterrupted duration of 258 seconds (Table 1).

Clinical studies have shown that humans aged 60–80 years have diminished autonomic cardiovascular responses [13, 14]. We hypothesized that the extrinsic autonomic nervous system, including innervation from the brain and spinal cord, exerts inhibitory control of the intrinsic autonomic nervous system preventing the intrinsic nerves and ganglia on the heart from becoming independently hyperactive. On the other hand, denervation [15] or disconnection of the extrinsic from the intrinsic innervation [16] induces a hyperactive state of the intrinsic system with the development of AF. We have demonstrated previously that stimulation of the vagosympathetic trunks to simulate tonic levels of activation can prevent and/or suppress AF induced by hyperactive remodeling of the ganglionated plexi of the intrinsic autonomic nervous system. In the dog heart, AF has been shown to be directly associated with excessive release of cholinergic and adrenergic neurotransmitters, the former shortening the refractory period and the latter inducing triggered firing [17, 18]. In our previous study using ACh infusions in the rabbit with enhanced activation of *β*2-adrenergic receptor, sustained atrial tachycardias were consistently evoked [10]. There is evidence in the present study for an increased level of sympathetic tone in the old rabbits whose mean heart rate was 200/min compared to the young rabbits with a rate of 160/min. As noted from our previous dog studies, ACh can activate neurons, which cause release of their neurotransmitters, in ganglionated plexi of the intrinsic autonomic nervous system [19]. These neurons have been shown to colocalize vesicles containing both cholinergic and adrenergic neurotransmitters [20], both of which are the major ingredients for initiation and maintenance of AF.

One of the first questions which arises when considering susceptibility to AF in old animals is the presence of atrial fibrosis. Whether there is a direct cause or effect relationship between atrial fibrosis and AF is still open to question. A histological study of lone AF patients found that 25% of patients did not have atrial fibrosis [21]. On the other hand, both clinical [22] and experimental [23] studies have associated atrial fibrosis development with structural heart disease or changes due to long term rapid ventricular pacing to induce heart failure, respectively. In the present study, we found no significant fibrosis in the atria of either old or young rabbits. However, our histological sections clearly showed moderate to severe interstitial fibrosis in the ventricles of the old rabbits but not in the young rabbits. Similar results were reported by Lee et al. [12] in old rabbit hearts, and, after providing several explanations such as 3-year-old rabbits may not be old enough (our rabbits were 4–6 years of age), they concluded that “atrial fibrosis may not play an important role in the induction of AF in rabbits with no significant structural heart disease. In addition, the increased incidence of AF as age increased may not solely be explained by atrial fibrosis.” It should be mentioned that an experimental aged rat model did show AF associated with fibrosis [2]. This difference may be related to species differences.

Appropriate animal models for AF are problematic. Issues include changes in innervation, size of the heart, and differences in aging. Recent pressures in the United States and elsewhere have led to significant restrictions on use of the common canine model. Substitution with small rodents is difficult owing to their small cardiac size and notable differences in biological function from humans. These difficulties are even greater when there is a need to study the effect of aging. The telescoped life span of rodents is advantageous for aging research but may not reflect the pathophysiology of larger mammals. The present study presents evidence that the rabbit may represent a convenient intermediate species wherein such studies of aging are possible within a reasonable time frame and the pathophysiological changes in the heart are not insurmountable.

## 5. Conclusions

Using a relatively new experimental model, we have demonstrated that rabbits 4–6 years of age have an increased susceptibility to develop AF compared to the young rabbits. This was associated with evidence supportive of increased autonomic activity and was induced by the combination of ACh and burst pacing. Atrial fibrosis was not observed in the atria and could be ruled out as a potential explanation for the greater propensity for AF in the old rabbits. This rabbit model presents several advantages for the study of immunological issues that affect atrial or ventricular tachyarrhythmias.

## Figures and Tables

**Table 1:** Comparison of atrial fibrillation induction in old versus young rabbits.

Rabbit	Baseline	10 *μ*M ACh	100 *μ*M ACh	1 mM ACh
Old #1	0	0	0	0
Old #2	<1^a^	1 and 4^a^	10	118
Old #3	<1	2	15	11
Old #4	0	0	11	3
Old #5	0	0	<1	681
Old #6	0	0	<2	960
Young #1	<1^a^	0	0	0
Young #2	<1	0	0	0
Young #3	0	0	0	2
Young #4	0	0	0	0
Young #5	0	0	0	0
Young #6	<1^a^	0	0	4
Young #7	<1	0	0	0
Young #8	0	0	0	3
Young #9	0	0	0	0

Numbers represent the duration (in seconds) of atrial fibrillation or atrial tachycardia induced.

^a^Atrial tachycardia.
